# Quantitative assessment of the effects of massive nucleic acid testing in controlling a COVID-19 outbreak

**DOI:** 10.1186/s12879-022-07816-2

**Published:** 2022-11-12

**Authors:** Wenlong Zhu, Yue Zhu, Zexuan Wen, Bo Zheng, Ao Xu, Ye Yao, Weibing Wang

**Affiliations:** 1 Shanghai Institute of Infectious Disease and Biosecurity, School of Public Health, Fudan University, Shanghai, 20032 China; 2 Department of Epidemiology, School of Public Health, Fudan University, 138 Yi Xue Yuan Road, Shanghai, 200032 China; 3 Key Laboratory of Public Health Safety of Ministry of Education, Fudan University, 138 Yi Xue Yuan Road, Shanghai, 200032 China

**Keywords:** COVID-19, Outbreak, Non-pharmaceutical intervention, Close contact tracing, Nucleic acid amplification testing, Vaccine, Branching process model, Transmission distance

## Abstract

**Background:**

From 20 July to 26 August 2021, local outbreaks of COVID-19 occurred in Nanjing City and Yangzhou City (Jiangsu Province, China). We analyzed the characteristics of these outbreaks in an effort to develop specific and effective intervention strategies.

**Methods:**

Publicly available data on the characteristics of the COVID-19 outbreaks in Jiangsu Province were collected. Logistic regression was used to assess the association of age and sex with clinical severity. Analysis of onset dates, generation time distributions, and locations were used to estimate the mean transmission distance. A branching process model was used to evaluate different management strategies.

**Results:**

From 20 July to 26 August 2021, 820 patients were diagnosed with COVID-19 in Jiangsu Province, with 235 patients (28.7%) from Nanjing, 570 (69.5%) from Yangzhou, and 15 (1.8%) from other cities. Overall, 57.9% of the patients were female, 13.7% were under 20 years-old, and 58.3% had moderate disease status. The mean transmission distance was 4.12 km, and closed-loop management of the area within 2.23 km of cases seemed sufficient to control an outbreak. The model predicted that the cumulative cases in Yangzhou would increase from 311 to 642 if the interval between rounds of nucleic acid amplification testing (NAAT) increased from 1 to 6 days. It also predicted there would be 44.7% more patients if the NAAT started 10 days (rather than 0 days) after diagnosis of the first case. The proportion of cases detected by NAAT would increase from 11.16 to 44.12% when the rounds of NAAT increased from 1 to 7 within 17 days. When the effective vaccine coverage was 50%, the outbreak would be controlled even when using the most relaxed non-pharmaceutical interventions.

**Conclusions:**

The model predicted that a timely closed-loop management of a 2.23 km area around positive COVID-19 cases was sufficient to control the outbreak. Prompt serial NAAT is likely to contain an outbreak quickly, and our model results indicated that three rounds of NAAT sufficiently controlled local transmission.

*Trial registration* We did not involve clinical trial.

**Supplementary Information:**

The online version contains supplementary material available at 10.1186/s12879-022-07816-2.

## Background

A new wave of Coronavirus disease 2019 (COVID-19) cases that were caused by new variants led to sporadic epidemics in many cities across China, including Ruili [1], Shanghai [2], and Shijiazhuang [3]. Although such epidemics were always contained within about 1 month, their sizes varied considerably. In particular, the number of total incident cases in a single city ranged from just a few to several hundred [4]. This indicates that even though recommended interventions were implemented, they may have been inappropriate. A sudden outbreak may lead to insufficient and delayed response, or even excessive response.

Massive nucleic acid amplification testing (NAAT), contact tracing, closed-loop management (i.e., management of the street and county where the patient lived) can successfully suppress local transmission, and these methods were implemented widely [5–7]. Severe acute respiratory syndrome coronavirus 2 (SARS-CoV-2), the causative virus of COVD-19, can be spread by infected individuals who are asymptomatic, presymptomatic, and symptomatic, so a large proportion of latent cases who are missed by contact tracing can be detected by massive NAAT [8]. However, infected cases will also be missed by NAAT if they are tested during the early stage of infection, when the viral load is below the limit of detection [9, 10]. Although comprehensive large-scale NAAT strategies support China’s sustained containment of COVID-19, the costs required for massive NAAT and quarantine are major challenges for society and the government. To achieve effective epidemic control and reduce socioeconomic costs, three key questions must be addressed. First, how many rounds of massive NAAT should be implemented? Second, what schedule of the massive NAAT should be performed to control the outbreak? Third, what criteria should be used for defining the populations who should be kept in closed-loop management?

These interventions were evaluated based on real-world epidemic data from a COVID-19 outbreak occurred in Nanjing City and Yangzhou City (Jiangsu Province, China). On 20 July 2021, a local outbreak of COVID-19 caused by imported cases occurred in Nanjing (Jiangsu Province). Within 2 weeks, the epidemic spread rapidly to 26 cities in 11 provinces and municipalities, and it was contained by late August 2021. Yangzhou, also in Jiangsu Province and adjacent to Nanjing, reported more than 500 cases. Almost all of the cases in Jiangsu province are reported from those two cities. Both cities implemented representative interventions, such as massive NAAT, closed-loop management, and other public health and social interventions (e.g., suspension of public transportation, closing of entertainment places, etc.). On 21 July, Lukou Street in Nanjing, the locus of the initial outbreak, was placed in lock-down. Since then, three rounds of massive NAAT were conducted in Nanjing [11]. On 31 July, a closed-loop approach was implemented in residential communities where cases were reported in Yangzhou. Three days later, a closed-loop approach was implemented in all residential communities in central areas (Hanjiang District and Guangling District). In addition, between 28 July and 12 August, seven rounds of massive NAAT were performed in Yangzhou [12].

In this study, we used publicly available data to analyze the characteristics of the recent local outbreak of COVID-19 in Nanjing and Yangzhou. In particular, we constructed a branching process model to simulate the spread and transmission of COVID-19, and assessed the impact of massive NAAT when it had different schedules, number of rounds, and start times. We also estimated the mean dispersal distance between transmission-linked cases to provide reference for closed-loop management.

## Methods

### Data collection

Data on the characteristics of COVID-19 infections in Nanjing and Yangzhou from 20 July to 26 August 2021 were from daily reports released by the Health Commission of Nanjing (http://wjw.nanjing.gov.cn/) and Yangzhou (http://wjw.yangzhou.gov.cn/). These publicly available data included sex, age, district of residence, dates of key epidemiological parameters (confirmed diagnosis, quarantine/isolation, and first positive NAAT), and information on close contacts of all cases. All data were extracted and entered into a structured database. Patients were diagnosed according to the Diagnosis and Treatment Protocol for Coronavirus Pneumonia (Trial Version 8) from the National Health Commission of China.

### Statistical analysis

All patients with infections were included in the statistical analysis. The geographical distribution of all patients and epidemic curves based on the date of confirmed diagnoses were plotted. A Wilcoxon-test was used to analyze the difference in the mean age of cases from Nanjing and Yangzhou. A χ^2^ test or Fisher’s exact test was used to compare other characteristics of these two groups. A univariate logistic regression model was used to study the association of age groups and sex with clinical severity (mild vs. moderate).

The geographical distribution of COVID-19 cases was presented using ArcGIS version 10.5 (Environmental Systems Research Institute, Inc.). The base layer of maps was based on the China map with review number of GS2016-1595 and unmodified, available open source: https://www.ngcc.cn/ngcc/html/1/391/392/16114.html. Statistical analyses were performed using R software version 4.0.2. The results of the statistical tests were two-sided, and a *P* value below 0.05 was considered significant. Odds ratios (ORs) with 95% confidence intervals (95% CIs) were also determined.

### Estimating mean distances between cases

The mean transmission distance between cases was estimated using a method developed by Salje et al., in which data for onset date, generation time distribution, and location were used to estimate the mean transmission distance [13]. A pair of cases occurring at time points t_1_ and t_2_ was separated by a variable number of transmission events (*θ*). The distance between sequential cases in a transmission chain (i.e., *θ* = 1) was characterized by a transmission kernel, which was the probability distribution function of all transmission distances. The assumptions were that transmission events were independent of each other, each infected individual had a single infector, and the distance between pairs of cases depended on the number of transmission events that separated them. Although contract tracing data were only available for a limited number of individuals, the mean distance between all case pairs could be estimated as follows:

$${\mu }_{t}\left({t}_{1},{t}_{2}, {\mu }_{k},{\sigma }_{k}\right)= \sum_{i}w\left(\theta =i,{t}_{1},{t}_{2}\right)\cdot {\mu }_{a}\left(\theta =i, {\mu }_{k},{\sigma }_{k}\right),$$

where ${\mu }_{t}\left({t}_{1},{t}_{2}, {\mu }_{k},{\sigma }_{k}\right)$ is the mean distance separating all pairs of cases, in which one occurs at t_1_ and the other at t_2_; ${\mu }_{a}\left(\theta , {\mu }_{k},{\sigma }_{k}\right)$ is the mean distance between pairs of cases separated by *θ* transmission events, in which the transmission kernel has mean ${\mu }_{k}$ and standard deviation ${\sigma }_{k}$; and $w\left(\theta ,{t}_{1},{t}_{2}\right)$ are the weights representing the proportions of case pairs that occur at t_1_ and t_2_ that are separated by *θ* transmission events.

The weights, $w\left(\theta ,{t}_{1},{t}_{2}\right)$, were estimated using a method based on the Wallinga–Teunis matrix [14] that calculates the probability that a pair of cases occurring at times t_1_ and t_2_ were separated by *θ* transmission events.

The mean distance ${\mu }_{a}\left(\theta , {\mu }_{k},{\sigma }_{k}\right)$ was assumed to have an approximately normal distribution based on the central limit theorem, and the mean ${\mu }_{k}$ and the standard deviation ${\sigma }_{k}$ were equal. Thus, ${\mu }_{a}\left(\theta , {\mu }_{k},{\sigma }_{k}\right)$ became:

$${\mu }_{a}\left(\theta , {\mu }_{k},{\sigma }_{k}\right)\approx 0.5\cdot \mu k\sqrt{2\pi i}$$

A weighted average estimate across all combinations of t_1_ and t_2_ is then:

$${\widehat{\mu }}_{k}={\widehat{\sigma }}_{k}=\frac{1}{{\sum }_{i}{\sum }_{j}{n}_{ij}}\sum_{i}\sum_{j}\frac{2\bullet {\mu }_{t}^{obs}\left({t}_{1},{t}_{2}\right)\bullet {n}_{ij}}{{\sum }_{k}\widehat{w}\left(\theta =k,{t}_{1},{t}_{2}\right)\bullet \sqrt{2\pi k}} ,$$

where ${\mu }_{t}^{obs}\left({t}_{1},{t}_{2}\right)$ is the observed mean distance between cases occurring at the two times and ${n}_{ij}$ is the number of case pairs in which one case occurred at time i and another at time j.

After transmission distance information was extracted from real-world data, it was assumed that the transmission distance distribution followed a typical power law distribution, in which the key parameter, λ, could be calculated by the mean. Thus, the transmission distance distribution was constructed and the threshold corresponding to an effective reproduction number (R_eff_) below 1 was extracted.

### Branching process model structure

**Fig. 1:**
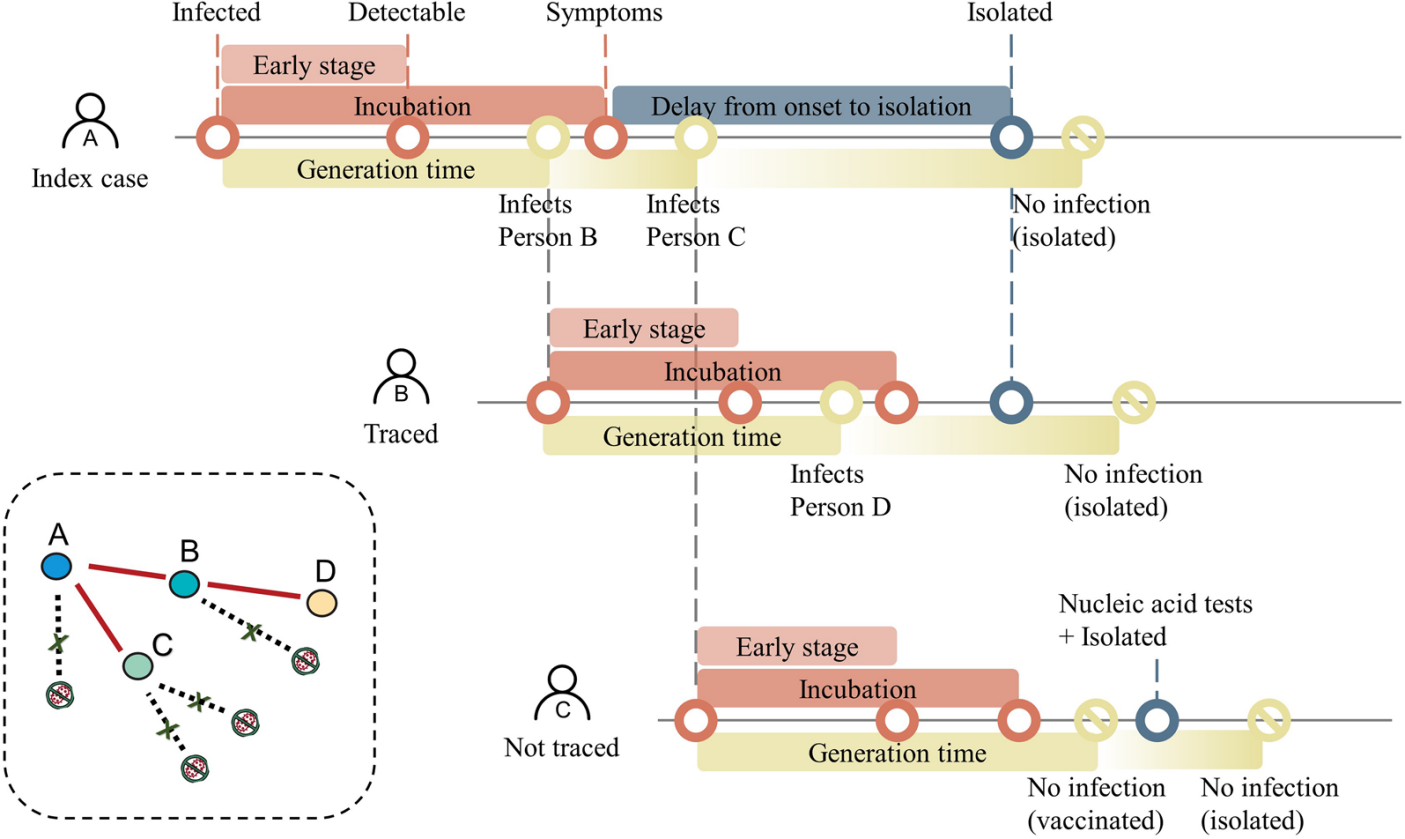
Hypothetical example of the simulation, which starts with the infection of the index case (A)

A branching process model was implemented as a Markov chain [15]. In particular, the number of potential secondary cases from each individual followed a negative binomial distribution with a mean equal to the R_eff_, which varied around 5 and had a SARS-like overdispersion (k = 0.16) [16]. Each potential new infection was assigned an exposure time from the generation time distribution. Secondary cases only occurred if they were ineffectively vaccinated and their infectors were not isolated at the time of infection. As a hypothetical example (Fig. 1), an infected person (case A) could potentially produce three secondary infections, but only two transmissions (case B and case C) occurred before isolation. Although case B was traced, he/she still infected case D. Case C was not traced, but was detected by NAAT and was then isolated.

All infected cases (symptomatic and asymptomatic) can be identified through contact tracing with provability ρ, or by NAAT with a sensitivity of 70%. It was assumed that a case missed because of a false negative during one round of NAAT would be identified in the subsequent round. In addition, an infected case would also be missed by the NAAT if the examination was during the early stage of infection, when the viral load is low [9, 10]; the early stage was drawn from a normal distribution with a mean of 3 days. Symptomatic cases were detected after symptom onset with a delay drawn from a Weibull distribution, with scale parameter of 5 days and a shape parameter of 2.5 days. All infected cases that were detected were isolated immediately, but transmission could not be prevented before isolation. Isolation was assumed to be 100% effective in preventing further transmission. In addition, each case had an independent probability of being asymptomatic, in which detection cannot occur by self-report. The subclinical cases and symptomatic cases were assumed to have the same transmission probabilities.

### Model parameters and scenarios

The first 24 days in Nanjing and the first 30 days in Yangzhou were used to simulate the outbreaks and obtain the number of initial cases, the effective vaccine coverage, and the probability of being traced. The results of Yangzhou were described in the main text, and the results of Nanjing were presented in the Additional file 1: Additional materials as sensitivity analysis.

In Nanjing, there were 22 asymptomatic cases and 235 symptomatic cases. The number of asymptomatic cases was not reported in Yangzhou. Thus, the probability of being asymptomatic was assumed to be 8.56% in both cities. Three rounds of massive NAAT were implemented in central areas of Nanjing on 21, 25, and 28 July [11], corresponding to 1, 5, and 8 days after the first confirmed case. Seven rounds of massive NAAT were implemented in central areas of Yangzhou on 28 July, 1, 5, 7, 9, 11, and 12 August 2021 [12], corresponding to 0, 4, 8, 10, 12, 14, and 15 days after the first confirmed case. The sensitivity of massive NAAT (70%) was based on a previous study [17].

The incubation period for each case was drawn from a log-normal distribution with scale parameter of 4.89 days and a shape parameter of 9.71 days [18]. The corresponding generation time for each case was then drawn from a skewed-normal distribution, with the mean of the distribution set to the incubation period for that case. This sampling approach ensured that the generation time and incubation period for each case were correlated and biologically plausible, because a case is likely to be more infectious soon after the time of onset [19].

Multiple scenarios were examined in which the massive NAAT had different intervals between tests, different number of rounds, and different start times. The interval between consecutive rounds of NAAT varied from 1 to 10 days, the number of rounds of NAAT varied from 1 to 8, and the start interval between first round of NAAT and the first confirmed case varied from 1 to 10 days. The descriptions of all parameters and their values are in Additional file 1: Table S1 [9–12, 15, 17, 18]. All simulations were performed using R software version 4.0.2.

## Results

**Fig. 2:**
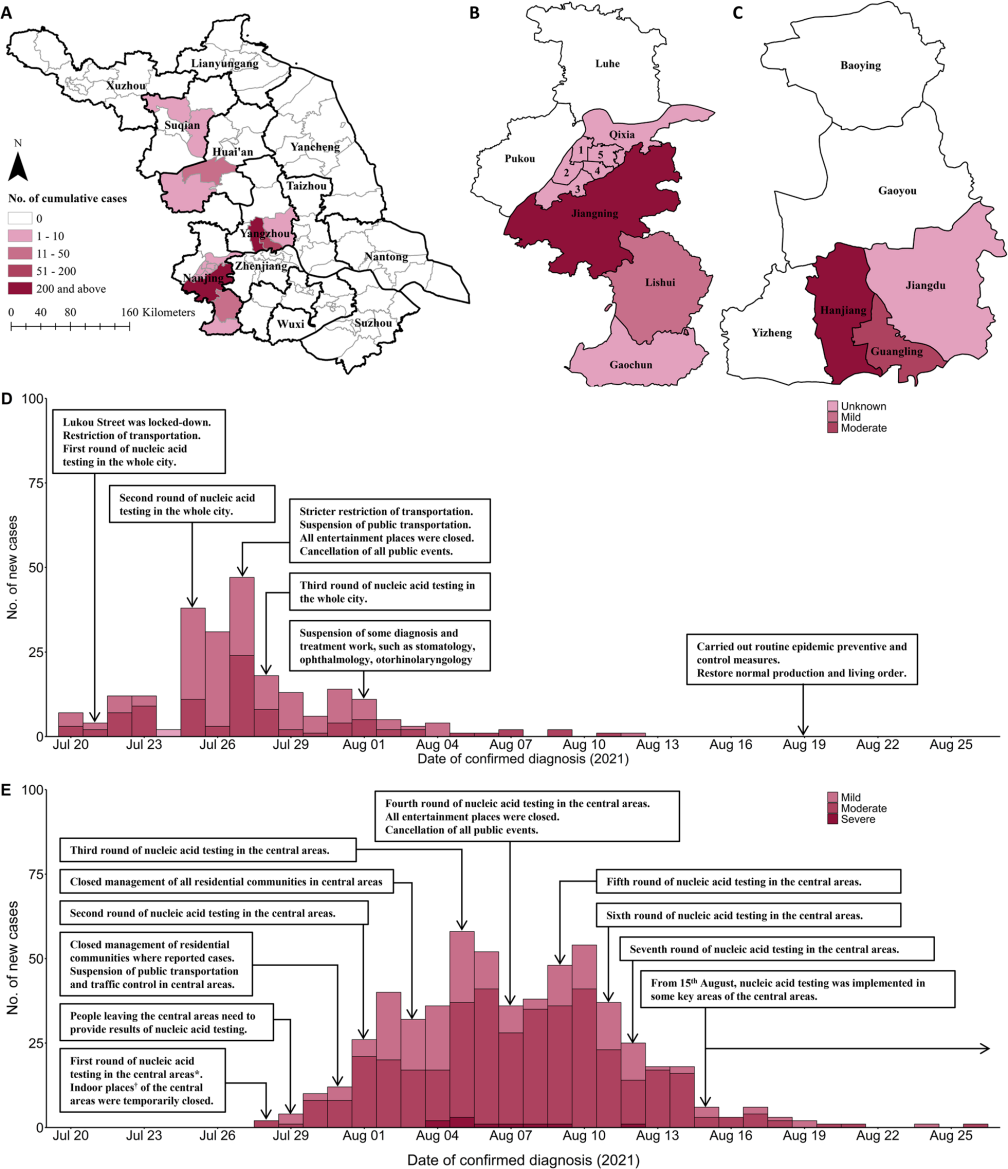
Geographic distribution of confirmed COVID-19 cases in Jiangsu Province (**A**), Nanjing City (**B**), and Yangzhou City (**C**), and number of new confirmed daily cases in Nanjing City (**D**) and Yangzhou City (**E**). The numbers in **B** refer to Gulou District (1), Jianye District (2), Yuhuatai District (3), Qinhuai District (4), and Xuanwu District (5). *Central areas included Hanjiang District, Guangling District, economic and technological Development Zone, and ecological science and technology district. ^†^Indoor places included indoor entertainment places, indoor stadiums, training institutions, religious places, bathing centers, etc. The base layer of maps (**A–C**) was based on the China map with review number of GS2016-1595 and unmodified, available open source: https://www.ngcc.cn/ngcc/html/1/391/392/16114.html

From 20 July to 26 August 2021, there were 820 COVID-19 patients in Jiangsu Province. The epidemic began in Nanjing, with 235 infected patients, and this was followed a second outbreak of 570 patients in Yangzhou. The outbreak epicenter in Nanjing was the Jiangning District and the outbreak epicenter in Yangzhou was the Hanjiang District (Fig. 2). Among the 820 COVID-19 patients, 57.9% were female, the median age was 47.6 years, and most patients (57.1%) were centrally quarantined before diagnosis (Table 1).

**Table 1:** Characteristics of patients who had laboratory-confirmed COVID-19 in Jiangsu Province

Characteristic	Overall	Other cities	Nanjing	Yangzhou	*P* value^#^
**All patients**	820 (100.0)	15 (1.8)	235 (28.7)	570 (69.5)	
**Sex**					0.573
Male	345 (42.1)	9 (60.0)	94 (40.0)	242 (42.5)	
Female	475 (57.9)	6 (40.0)	141 (60.0)	328 (57.5)	
Age, years*	47.6 (21.5)	49.2 (15.2)	42.9 (17.6)	49.5 (22.7)	< 0.001
**Age group**					< 0.001
0–19	112 (13.7)	1 (6.7)	29 (12.3)	82 (14.4)	
20–39	161 (19.6)	2 (13.3)	50 (21.3)	109 (19.1)	
40–59	280 (34.1)	9 (60.0)	121 (51.5)	150 (26.3)	
60–79	230 (28.0)	3 (20.0)	33 (14.0)	194 (34.0)	
80 +	37 (4.5)	0 (0.0)	2 (0.9)	35 (6.1)	
**Central quarantine**					0.389
No	346 (42.2)	0 (0.0)	95 (40.4)	251 (44.0)	
Yes	468 (57.1)	9 (60.0)	140 (59.6)	319 (56.0)	
Unknown	6 (0.7)	6 (40.0)	0 (0.0)	0 (0.0)	
**Clinical severity**					< 0.001^†^
Mild	316 (38.5)	0 (0.0)	143 (60.9)	173 (30.4)	
Moderate	478 (58.3)	1 (6.7)	90 (38.3)	387 (67.9)	
Severe	10 (1.2)	0 (0.0)	0 (0.0)	10 (1.8)	
Unknown	16 (2.0)	14 (93.3)	2 (0.9)	0 (0.0)	

*Age is given as mean (SD) and all other values as N (%)

^#^*P* values were calculated for comparisons of Nanjing and Yangzhou

^†^ *P* value was calculated by Fisher's test after excluding patients with unknown clinical severity

**Fig. 3:**
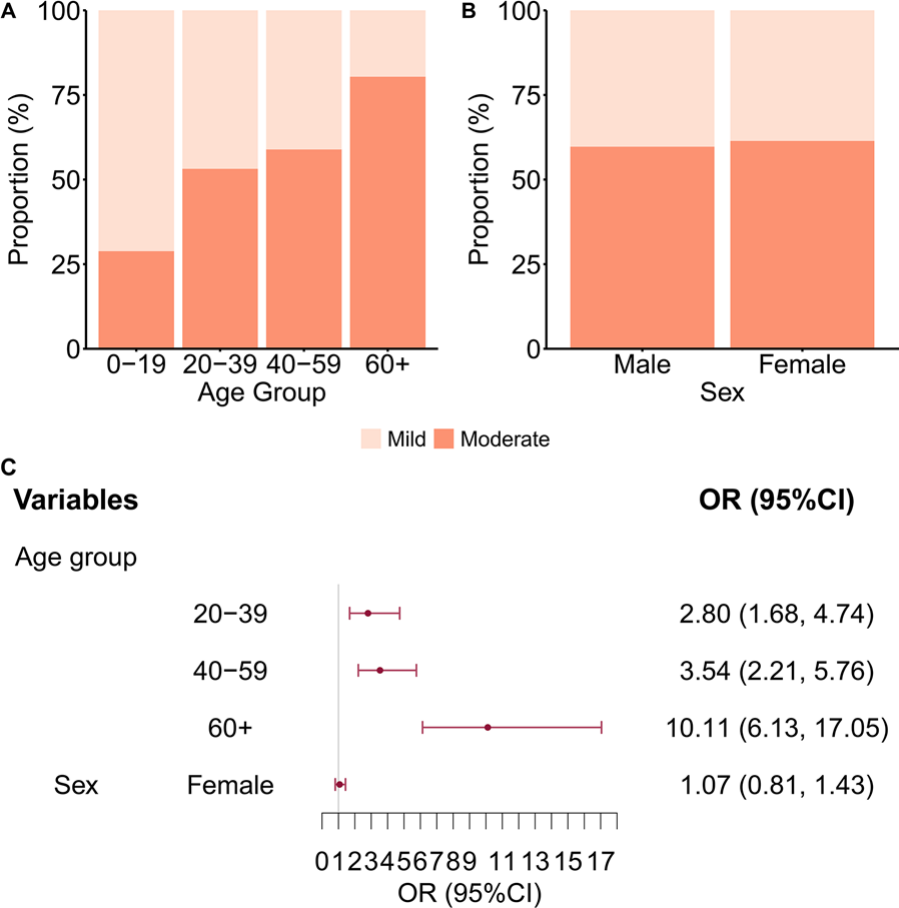
Univariate logistic regression analysis of the association of age groups (**A**) and sex (**B**) with clinical severity (mild or moderate) in patients from Jiangsu Province, and OR (95%CI) from the univariate logistic regression (**C**)

Among the 804 patients whose clinical severity was known, 60.70% had moderate severity and 39.3% had mild severity. The proportion of patients with moderate severity was 28.83% in the youngest age group, and 80.37% in the oldest age group (Fig. 3A). The proportion of patients with moderate severity was similar in males (59.70%) and females (61.41%; Fig. 3B). Univariate logistic regression indicted that patients in the oldest group (60+ years-old) were 10.11-fold (95%CI 6.13–17.05) more likely to develop moderate disease than those in the youngest age group (0–19 years-old). There was no statistical difference in risk for males and females (Fig. 3C). Stratification by city indicated the results were the same for Nanjing (Additional file 1: Fig. S1) and Yangzhou (Additional file 1: Fig. S2).

**Fig. 4:**
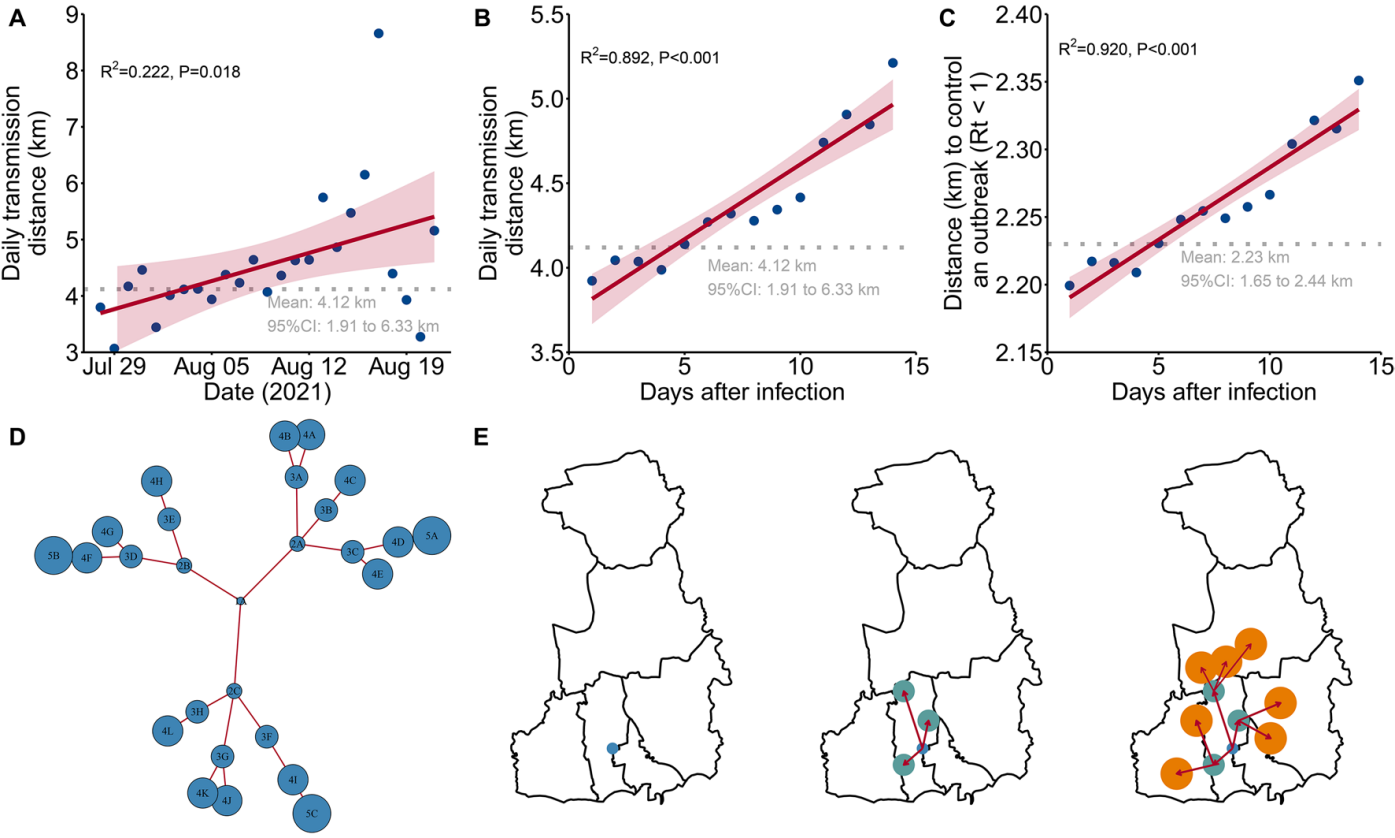
Daily transmission distance of the outbreak in Yangzhou City, showing the association between daily transmission distance and date (**A**), daily transmission distance and days after infection (**B**), and distance needed to control an outbreak and the days after infection (**C**). The grey dotted lines in **A**–**C** are the mean distances (4.12 km in **A** and **B**, 2.23 km in **C**). Assumed simple transmission chain based on analysis of data in Yangzhou, in which circles are sized according to the geographical distance from the index case (1A) (**D**). The geographical distribution of cases from the assumed simple transmission chain in **D**, in which the generation of cases is distinguished by color (**E**). The base layer of maps (**E**) was based on the China map with review number of GS2016-1595 and unmodified, available open source: https://www.ngcc.cn/ngcc/html/1/391/392/16114.html

The mean transmission distance per infected person in the outbreak of Yangzhou was 4.12 km (95%CI 1.91–6.33), and the daily transmission distance increased over time (*P* = 0.018, Fig. 4A). The daily transmission distance increased from 3.92 km at the time of infection to 5.21 km at 15 days after infection (*P* < 0.001, Fig. 4B). A closed-loop management showed that an area within 2.23 km (95%CI 1.65–2.43) of a case seemed sufficient to contain the outbreak (i.e., maintain R_eff_ below 1; Fig. 4C). The area of the closed-loop management increased over time (*P* < 0.001, Fig. 4C), indicating that early identification and control of a patient require a smaller scope of investigation and management. The daily transmission distance increased slightly over time; however, the spread of the disease and the area of closed-loop management both increased rapidly as the number of infected people increased (Fig. 4D, E). In Nanjing City, the mean transmission distance was 31.93 km (95%CI 12.43–51.42), and the mean distance to control an outbreak was 3.79 km (95%CI 4.54–3.86; Additional file 1: Fig. S3).

**Fig. 5:**
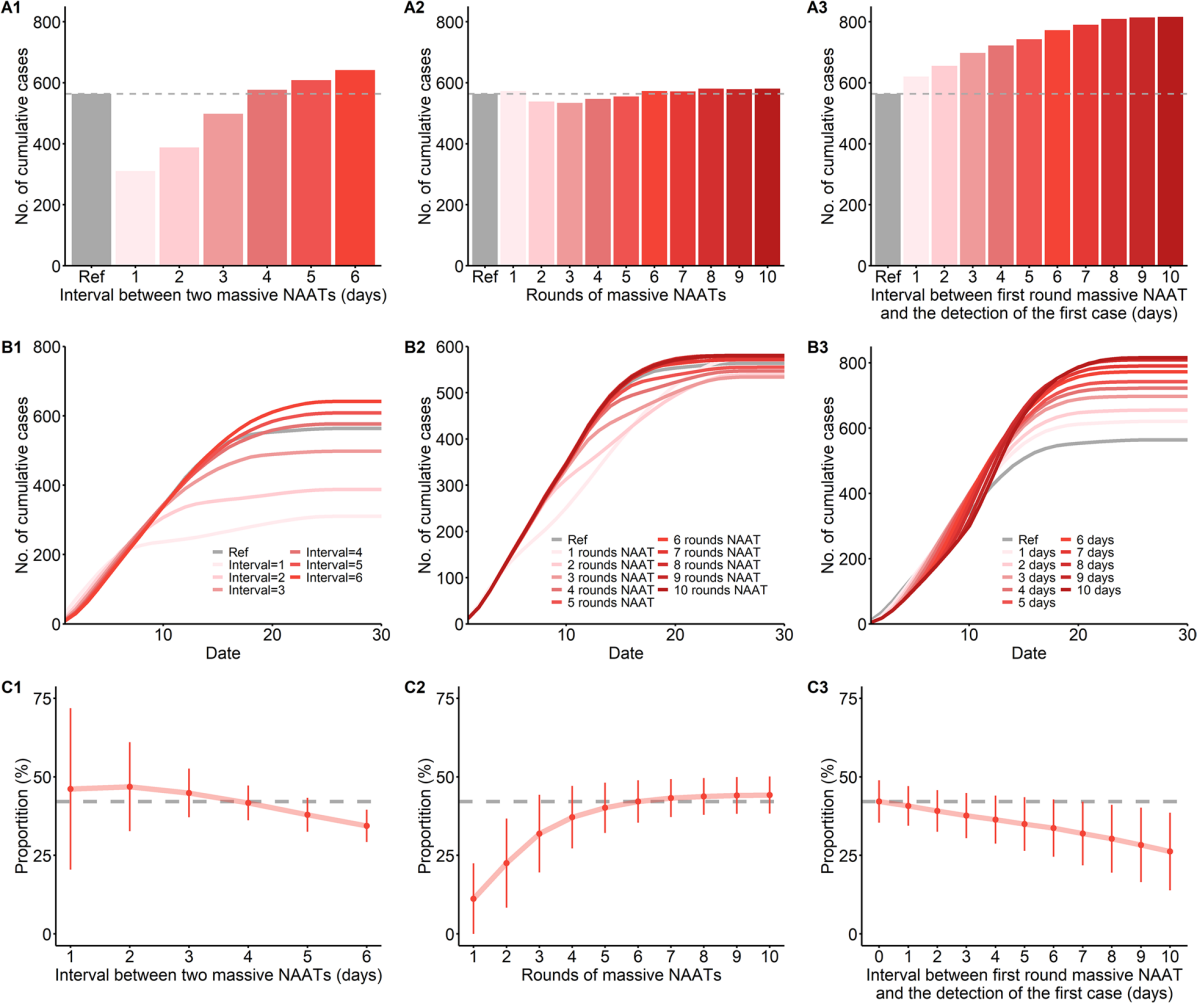
Number of cumulative cases (**A**, **B**) and percentage of cases found by massive NAAT (**C**) in Yangzhou City for different intervals of NAAT (**A1**, **B1**, **C1**), different number of rounds of NAAT (**A2**, **B2**, **C2**), and different start times of NAAT (**A3**, **B3**, **C3**). The error bars in **C** indicate 95% reference intervals

We used the branching process model to examine the effect of the frequency, number of rounds, and timing of NAAT on controlling the outbreak in Yangzhou City (Fig. 5). Relative to an NAAT interval of 4 days, the number of cumulative cases decreased by 44.88% when the interval was 1 day, decreased 31.21% when the interval was 2 days, and decreased 11.60% when the interval was 3 days. The cumulative number of cases increased from 564 to 641 when the interval increased from 4 to 6 days (Fig. 5A1). Use of more rounds of the massive NAAT seemed to have little effect on the number of cumulative cases (Fig. 5A2). However, the cumulative cases increased from 564 to 816 when the massive NAAT start time increased from 0 to 10 days after detection of the first case (Fig. 5A3). A shorter interval between NAAT rounds and an earlier NAAT start time led to identification of more cases early during the epidemic (Fig. 5B1, B3). When the rounds of NAAT were more than 2, NAAT performed similar for early identification of cases (Fig. 5B2). In Yangzhou City, we estimated that 42.13% of cases were found when the NAAT interval was 4 days, there were six rounds of NAAT, and the NAAT start time was 0 days after detection of the first case. The percentage decreased from 46.15 to 34.37% when the NAAT interval increased from 1 to 6 days (Fig. 5C1); the percentage increased from 11.16 to 44.12% when the number of NAAT rounds increased from 1 to 7 (Fig. 5C2). However, increasing the number of rounds of NAAT to more than 5 had a small effect on the percentage of cases identified. The percentage of cases found by massive NAAT decreased from 42.13 to 26.23% when the NAAT started on day 10 rather than day 0 after detection of the first case (Fig. 5C3). These same trends occurred in simulation of the outbreak of Nanjing City (Additional file 1: Fig. S4). In the outbreak of Nanjing City, an estimated 34.2% of cases were found by massive NAAT when the NAAT interval was 3 days, there were three rounds of NAAT, and the NAAT start time was 1 day after detection of the first case.

**Fig. 6:**
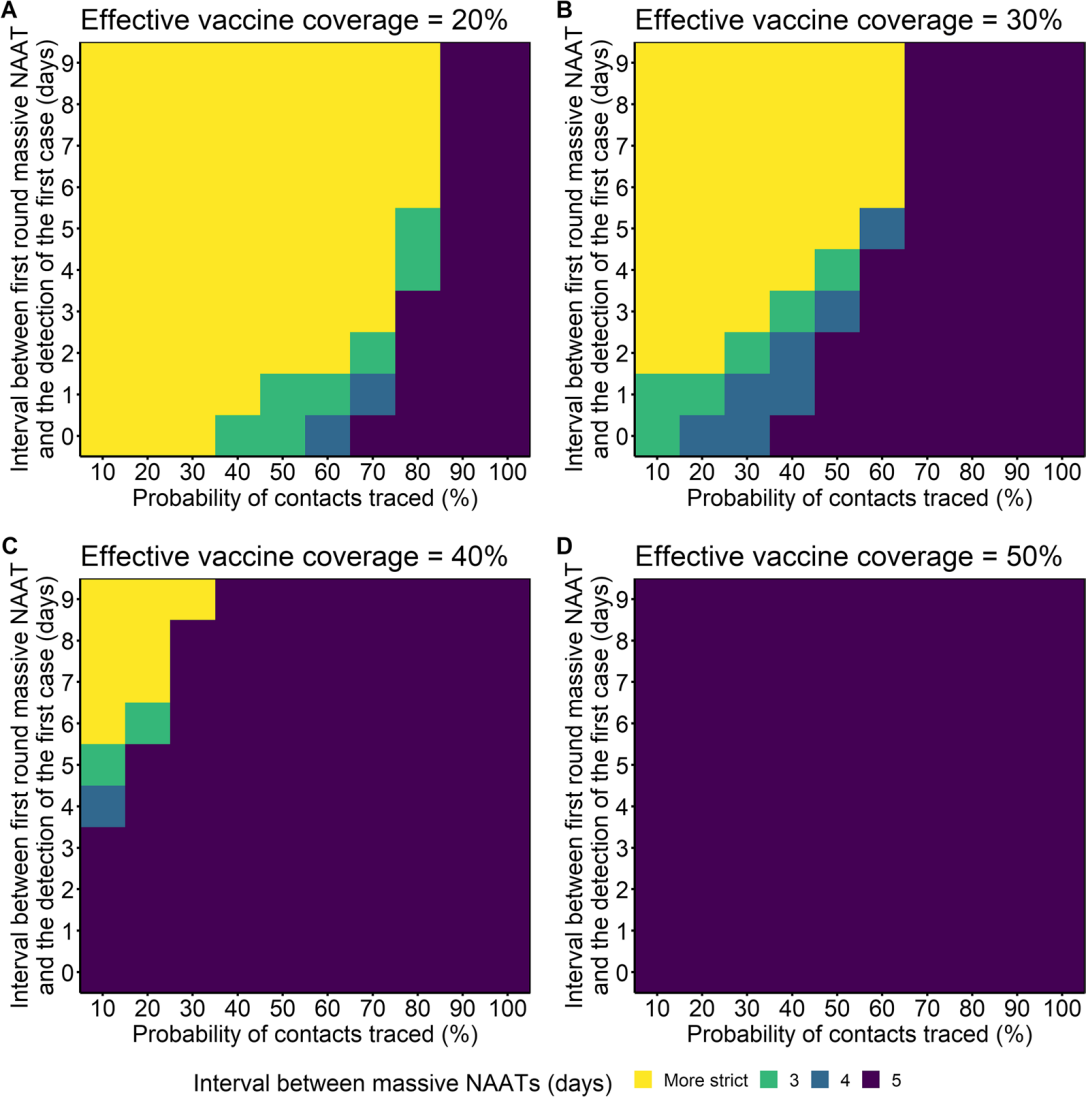
The combined effects of vaccine coverage (20% in A, 30% in B, 40% in C and 50% in D), close contact tracing, and massive NAAT on limiting the cumulative number of cases in Yangzhou City to 570. Each scenario had four rounds of NAAT

If the effective vaccine coverage was 20%, more than 90% of cases were traced, and the interval between NAAT rounds was 5 days, even a late initiation of the massive NAAT did not increase the cumulative number of cases above 570 (Fig. 6A). However, when the effective vaccine coverage was 20% and fewer than 90% of cases were traced, more extensive NAAT or other non-pharmaceutical interventions (NPIs) were needed to control the outbreak. When effective vaccine coverage increased from 30 to 50%, outbreaks were also controlled when using the most relaxed NAAT, even if the probability of contact tracing decreased from 70 to 10% (Fig. 6B–D). Once the effective vaccine coverage reached 30%, even when the probability of contact tracing was only 10%, the outbreak was also controlled by implementation of massive NAAT when the interval between NAAT rounds was 3 days, and NAAT started 0 or 1 day after detection of the first case (Fig. 6B). If effective vaccine coverage was 50% and the interval between NAAT rounds was 5 days, there were fewer than 570 cumulative cases regardless of when NAAT started (Fig. 6D). We found the same effects in Nanjing City (Additional file 1: Fig. S5). In particular, if the effective vaccine coverage reached 40%, the outbreak was controlled even when using the most relaxed NPIs.

## Discussion

The COVID-19 outbreak in Jiangsu Province during 2021 had 820 symptomatic patients, and a greater percentage of these patients were female, consistent with previous studies [3, 20]. However, relative to previous studies [20–22], more of our patients were 0–19 years-old, similar to the outbreak in Shijiazhuang (Hebei Province) [3]. Children and adolescents who are asymptomatic or only mildly symptomatic tend not to attract much medical attention during a large or widespread epidemic [20, 23, 24], although small localized outbreaks in which everyone receives at least one round of NAAT [3, 11, 12] increases detection of infections in young people. Consistent with previous studies [24–26], our results also suggested that the risk of severe illness increased with age. Children and adolescents are usually infected during the second or third generation of infections, and the virus may be less virulent at these times [27]. The severity of COVID-19 also correlates with the neutrophil-to-lymphocyte ratio (NLR), which increases with age [28].

The prompt response and use of a series of NPIs led to control of the outbreak in Nanjing City within 23 days and in Yangzhou City within 29 days. Sixty percent of the patients in Nanjing and 56% of the patients in Yangzhou were identified by close contact tracing, and 34% (Nanjing) and 42% (Yangzhou) were detected by the massive NAAT. This indicates that strict NPIs for small outbreaks can detect most cases. Although close contact tracing plays a significant role in controlling virus transmission [15, 29], reporting accuracy, adherence, coverage, and timing can influence the effectiveness of contact tracing [29]. We found that when the mean transmission distance was 4.12 km, a closed-loop management of the area within 2.23 km of the infected patient was sufficient to maintain R_eff_ value below 1. These results provide a reference for the use of contact tracing and closed-loop management, and may be useful for improving the effectiveness of NPIs in future outbreaks. Notably, we determined the distance for closed-loop management when considering other interventions (massive NAAT, vaccination, etc.). Without these other NPIs and vaccination, it is likely that the area needed for closed-loop management would be much larger.

Massive NAAT is important for sustaining the containment of COVID-19 [30], and may partially make up for inefficiencies in close contact tracing. For a given number of rounds of NAAT, a shorter interval between NAAT leads to fewer cases. In addition, more rounds of NAAT lead to fewer cumulative cases over time. Importantly, our model simulation suggested that implementing more than 5 rounds of NAAT provided little benefit in detection of new cases. Considering the cost of NAAT, we suggest that 3–5 rounds of massive NAAT is appropriate. Our results also suggested that early initiation of NAAT reduced the number of cumulative cases, and when NAAT was initiated 7 days or more after identifying the first case, it had little impact on the number of cumulative cases.

The combination of close contact tracing and NAAT plays an essential role in controlling COVID-19 outbreaks. However, these NPIs have some limitations, such as high cost, inconveniences for the population, and limited efficacy. An effective vaccine is the major tool for prevention of further morbidity and mortality from COVID-19 [31]. Previous research indicated that if high vaccine coverage is achieved, then COVID-19 outbreaks can remain under control even when NPIs are lifted [32, 33]. The results of the present study confirmed that if the effective vaccine coverage reached 50%, then outbreaks would remain contained even when using the most relaxed methods for close contact tracing and NAAT. To control the spread of highly transmissible variants, it is necessary to accelerate the vaccination campaign [33].

This study had some limitations. First, we were unable to completely elucidate the transmission chain of Nanjing City, and this prevented use of complete transmission chains in the model. Although the mean transmission distance can be calculated using a simulated transmission chain, this distance would be more accurate when based on actual data. Second, the accuracy of the simulation was limited due to the lack of certain details, such as age-specific contact tracing data. Use of more detailed and specific parameters could allow the branching process model to simulate more diverse scenarios. Third, this study was based on delta variant outbreaks in Nanjing and Yangzhou. Application of our findings to outbreaks caused by high transmissibility variants or those occurring in larger cities should be considered with caution.

## Conclusions

The rapid adoption and a high frequency of NAAT led to fewer cases of COVID-19, and 3 rounds of massive NAAT appeared sufficient to control an outbreak in cities such as Nanjing and Yangzhou. Based on a mean transmission distance of 4.12 km, the prompt closed-loop management of an area of 2.23 km around a positive case was sufficient to contain an outbreak. In populations with high effective vaccine coverage, the combination of effective close contact tracing and massive NAAT may help to rapidly minimize outbreaks.

## Supplementary Information

**Additional file 1: Table S1.** Initial baseline values and distributions, and values used in different scenarios of the branching process model. **Figure S1.** Univariate logistic regression analysis of the association of age groups (**A**) and sex (**B**) with clinical severity (mild or moderate) in patients from Nanjing, and OR (95%CI) from the univariate logistic regression (**C**). **Figure S2.** Univariate logistic regression analysis of the association of age groups (**A**) and sex (**B**) with clinical severity (mild or moderate) in patients from Yangzhou, and OR (95%CI) from the univariate logistic regression (**C**). **Figure S3.** Daily transmission distance of the outbreak in Nanjing City, showing the association between daily transmission distance and date (**A**), daily transmission distance and days after infection (**B**), and distance used to control the outbreak and days after infection. The horizontal grey dotted lines are mean distances (31.93 km in **A** and **B**, 3.79 km in **C**). **Figure S4.** Number of cumulative cases (A and B) and percentage of cases found by massive NAAT (C) in Nanjing City for different intervals of NAAT (A1, B1, C1), different number of rounds of NAAT (A2, B2, C2), and different start time of NAAT (A3, B3, C3). The error bars in C, indicate 95% reference intervals. **Figure S5.** Combined effect of vaccine coverage, close contact tracing, and massive NAAT on limiting the cumulative number of cases in Nanjing City to 235.

## Data Availability

The datasets used and/or analyzed during the current study are available from the corresponding author on reasonable request.

## Abbreviations

COVID-19: Coronavirus disease 2019
NAAT: Nucleic acid amplification testing
SARS-CoV-2: Severe acute respiratory syndrome coronavirus 2
NPI: Non-pharmaceutical intervention
95%CI: 95% Confidence interval
km: Kilometer
R_eff_: Effective reproduction number

## References

[1] Xiangyu Y, Litao C, Zekun W, Linhui H, Zhongwei J, Bo Z (2021). Exploring the bridge cases’ role in the transmission of the SARS-CoV-2 Delta variant—Ruili city, Yunnan province, China, July–September 2021. China CDC Wkly.

[2] Shanghai Municipal Health Commission. 2022. https://wsjkw.sh.gov.cn/. Accessed 6 Jan 2022.

[3] Zhu W, Zhang M, Pan J, Yao Y, Wang W (2021). Effects of prolonged incubation period and centralized quarantine on the COVID-19 outbreak in Shijiazhuang, China: a modeling study. BMC Med.

[4] Lei Z, Kai N, Hongting Z, Xiang Z, Bixiong Y, Ji W (2021). Eleven COVID-19 outbreaks with local transmissions caused by the imported SARS-CoV-2 Delta VOC—China, July–August, 2021. China CDC Wkly.

[5] Lai S, Ruktanonchai NW, Zhou L, Prosper O, Luo W, Floyd JR (2020). Effect of non-pharmaceutical interventions to contain COVID-19 in China. Nature.

[6] Flaxman S, Mishra S, Gandy A, Unwin HJT, Mellan TA, Coupland H (2020). Estimating the effects of non-pharmaceutical interventions on COVID-19 in Europe. Nature.

[7] Teslya A, Pham TM, Godijk NG, Kretzschmar ME, Bootsma MCJ, Rozhnova G (2020). Impact of self-imposed prevention measures and short-term government-imposed social distancing on mitigating and delaying a COVID-19 epidemic: a modelling study. PLoS Med.

[8] Wiersinga WJ, Rhodes A, Cheng AC, Peacock SJ, Prescott HC (2020). Pathophysiology, transmission, diagnosis, and treatment of coronavirus disease 2019 (COVID-19): a review. JAMA.

[9] He X, Lau EHY, Wu P, Deng X, Wang J, Hao X (2020). Temporal dynamics in viral shedding and transmissibility of COVID-19. Nat Med.

[10] Kretzschmar ME, Rozhnova G, Bootsma MCJ, van Boven M, van de Wijgert J, Bonten MJM (2020). Impact of delays on effectiveness of contact tracing strategies for COVID-19: a modelling study. Lancet Public Health.

[11] Government of Nanjing Municipal. 2022. http://www.nanjing.gov.cn/zt/yqfk/index.html. Accessed 6 Jan 2022.

[12] Government of Yangzhou Municipal. 2022. http://yangzhou.gov.cn/. Accessed 6 Jan 2022.

[13] Salje H, Cummings DAT, Lessler J (2016). Estimating infectious disease transmission distances using the overall distribution of cases. Epidemics.

[14] Wallinga J, Teunis P (2004). Different epidemic curves for severe acute respiratory syndrome reveal similar impacts of control measures. Am J Epidemiol.

[15] Hellewell J, Abbott S, Gimma A, Bosse NI, Jarvis CI, Russell TW (2020). Feasibility of controlling COVID-19 outbreaks by isolation of cases and contacts. Lancet Glob Health.

[16] Lloyd-Smith JO, Schreiber SJ, Kopp PE, Getz WM (2005). Superspreading and the effect of individual variation on disease emergence. Nature.

[17] Zhang WT, Liu D, Xie CJ, Shen D, Chen ZQ, Li ZH (2021). Sensitivity and specificity of nucleic acid testing in close contacts of COVID-19 cases in Guangzhou. Zhonghua Liu Xing Bing Xue Za Zhi.

[18] Zhang M, Xiao J, Deng A, Zhang Y, Zhuang Y, Hu T (2021). Transmission dynamics of an outbreak of the COVID-19 Delta variant B.1.617.2—Guangdong province, China, May–June 2021. China CDC Wkly.

[19] Ge Y, Martinez L, Sun S, Chen Z, Zhang F, Li F (2021). COVID-19 transmission dynamics among close contacts of index patients with COVID-19: a population-based cohort study in Zhejiang province, China. JAMA Intern Med.

[20] Wu Z, McGoogan JM (2020). Characteristics of and important lessons from the coronavirus disease 2019 (COVID-19) outbreak in China: summary of a report of 72314 cases from the Chinese Center for Disease Control and Prevention. JAMA.

[21] Ladhani SN, Amin-Chowdhury Z, Davies HG, Aiano F, Hayden I, Lacy J (2020). COVID-19 in children: analysis of the first pandemic peak in England. Arch Dis Child.

[22] Gudbjartsson DF, Helgason A, Jonsson H, Magnusson OT, Melsted P, Norddahl GL (2020). Spread of SARS-CoV-2 in the Icelandic population. N Engl J Med.

[23] Mehta NS, Mytton OT, Mullins EWS, Fowler TA, Falconer CL, Murphy OB (2020). SARS-CoV-2 (COVID-19): what do we know about children? A systematic review. Clin Infect Dis.

[24] Dong Y, Mo X, Hu Y, Qi X, Jiang F, Jiang Z (2020). Epidemiology of COVID-19 among children in China. Pediatrics.

[25] Zhang JJ, Cao YY, Tan G, Dong X, Wang BC, Lin J (2021). Clinical, radiological, and laboratory characteristics and risk factors for severity and mortality of 289 hospitalized COVID-19 patients. Allergy.

[26] Verity R, Okell LC, Dorigatti I, Winskill P, Whittaker C, Imai N (2020). Estimates of the severity of coronavirus disease 2019: a model-based analysis. Lancet Infect Dis.

[27] Chen J, Zhang ZZ, Chen YK, Long QX, Tian WG, Deng HJ (2020). The clinical and immunological features of pediatric COVID-19 patients in China. Genes Dis.

[28] Brodin P (2021). Immune determinants of COVID-19 disease presentation and severity. Nat Med.

[29] Davis EL, Lucas TCD, Borlase A, Pollington TM, Abbott S, Ayabina D (2021). Contact tracing is an imperfect tool for controlling COVID-19 transmission and relies on population adherence. Nat Commun.

[30] Li Z, Liu F, Cui J, Peng Z, Chang Z, Lai S (2021). Comprehensive large-scale nucleic acid-testing strategies support China’s sustained containment of COVID-19. Nat Med.

[31] Hodgson SH, Mansatta K, Mallett G, Harris V, Emary KRW, Pollard AJ (2020). What defines an efficacious COVID-19 vaccine? A review of the challenges assessing the clinical efficacy of vaccines against SARS-CoV-2. Lancet Infect Dis.

[32] Leung K, Wu JT, Leung GM (2021). Effects of adjusting public health, travel, and social measures during the roll-out of COVID-19 vaccination: a modelling study. Lancet Public Health.

[33] Giordano G, Colaneri M, Di Filippo A, Blanchini F, Bolzern P, De Nicolao G (2021). Modeling vaccination rollouts, SARS-CoV-2 variants and the requirement for non-pharmaceutical interventions in Italy. Nat Med.

