# Supplementary material for: Quantitative assessment of the effects of massive nucleic acid testing in controlling a COVID-19 outbreak
Source: BMC Infect Dis. 2022 Nov 12;22:845. doi: 10.1186/s12879-022-07816-2 (PMC9652905; doi:10.1186/s12879-022-07816-2)
Supplement: Supplementary file 1 — Additional file 1: Table S1. Initial baseline values and distributions, and values used in different scenarios of the branching process model. Figure S1. Univariate logistic regression analysis of the association of age groups (A) and sex (B) with clinical severity (mild or moderate) in patients from Nanjing, and OR (95%CI) from the univariate logistic regression (C). Figure S2. Univariate logistic regression analysis of the association of age groups (A) and sex (B) with clinical severity (mild or moderate) in patients from Yangzhou, and OR (95%CI) from the univariate logistic regression (C). Figure S3. Daily transmission distance of the outbreak in Nanjing City, showing the association between daily transmission distance and date (A), daily transmission distance and days after infection (B), and distance used to control the outbreak and days after infection. The horizontal grey dotted lines are mean distances (31.93 km in A and B, 3.79 km in C). Figure S4. Number of cumulative cases (A and B) and percentage of cases found by massive NAAT (C) in Nanjing City for different intervals of NAAT (A1, B1, C1), different number of rounds of NAAT (A2, B2, C2), and different start time of NAAT (A3, B3, C3). The error bars in C, indicate 95% reference intervals. Figure S5. Combined effect of vaccine coverage, close contact tracing, and massive NAAT on limiting the cumulative number of cases in Nanjing City to 235. [file 12879_2022_7816_MOESM1_ESM.docx]

**Additional Materials for**

**Quantitative assessment of the effects of massive nucleic acid testing in controlling COVID-19 outbreaks**

Wenlong Zhu^1,2†^, Yue Zhu^2†^, Zexuan Wen^2^, Bo Zheng^2^, Ao Xu^2^, Ye Yao^2*^, Weibing Wang^1,2,3*^

1 Shanghai Institute of Infectious Disease and Biosecurity, School of Public Health, Fudan University, Shanghai 20032, China

2 Department of Epidemiology, School of Public Health, Fudan University, Shanghai 200032, China

3 Key Laboratory of Public Health Safety of Ministry of Education, Fudan University, Shanghai 200032, China

† These authors contributed equally to this work.

* Corresponding authors:

Dr. Ye Yao

School of Public Health, Fudan University, 138 Yi Xue Yuan Road, Shanghai 200032, China (e-mail: yyao@fudan.edu.cn).

Dr. Weibing Wang

Department of Epidemiology, School of Public Health & Key Laboratory of Public Health Safety (Ministry of Education), Fudan University, 138 Yi Xue Yuan Road, Shanghai 200032, China (e-mail: wwb@fudan.edu.cn).

**This file includes:**

Table S1

Figure S1 to Figure S5

Table S1. Initial baseline values and distributions, and values used in different scenarios of the branching process model.

| **Parameter** | **Initial values/**  **Distributions** | **Values in different scenarios** | **Source of initial values** |
| --- | --- | --- | --- |
| Delay between exposure and a positive test | Normal distribution,  Mean=3, SD=1 | - | Assumed, Ref [9,10] |
| Delay between onset and isolation | Weibull distribution,  Shape=2.5, Scale=5 | - | Assumed |
| Number of initial cases | Nanjing: 39  Yangzhou: 24 | - | Estimated |
| R_0_ for isolated cases | 0 | - | Assumed |
| R_0_ for non-isolated cases | Nanjing: 3.96  Yangzhou: 2.88 | - | Estimated |
| Intervals of NAAT | Nanjing: 4  Yangzhou: 3 | Nanjing: 1 to 10, difference=1  Yangzhou: 1 to 6, difference=1 | Ref [11,12] |
| Rounds of NAAT | Nanjing: 3  Yangzhou: 7 | Nanjing:8  Yangzhou:10 | Ref [11,12] |
| Start time of NAAT | Nanjing:21 July 2021, 1 day after the first case  Yangzhou: 28 July 2021, 0 days after the first case found | 0 to 10, difference=1 day after finding the first case | Ref [11,12] |
| Number of potential secondary cases | Negative binomial distribution,  Non-isolated, size=0.16  Isolated, size=1 | - | Ref [13] |
| Percentage of asymptomatic infections | 8.56% | - | Calculated |
| Generation time | Skew-Normal Distribution,  xi (location parameters) =onset of index cases, omega=2, alpha (k)=0.7 | - | Assumed, Ref [13] |
| Effective vaccine coverage | Nanjing: 23.89%  Yangzhou: 20.86% | 20%, 30%, 40%, 50% | Estimated |
| Probability of close contract tracing | Nanjing: 0.596  Yangzhou: 0.560 | 0.1 to 1, difference=0.1 | Calculated |
| Sensitivity of NAAT | 70% | - | Ref [15] |
| Incubation period | Log-normal distribution,  Meanlog=1.40, sdlog=0.41 | - | Ref [16] |

SD: standard deviation.


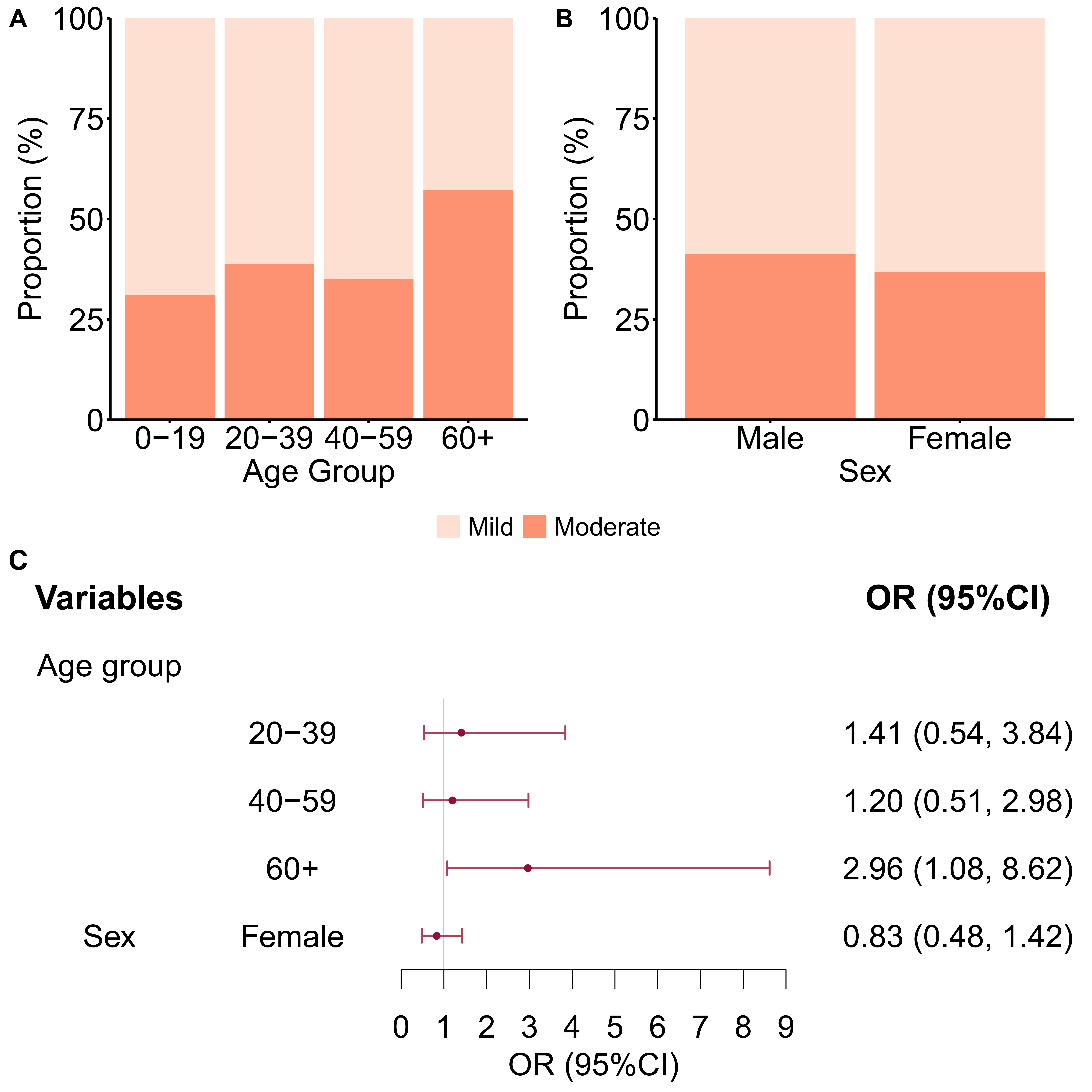
Figure S1. Univariate logistic regression analysis of the association of age groups (**A**) and sex (**B**) with clinical severity (mild or moderate) in patients from Nanjing, and OR (95%CI) from the univariate logistic regression (**C**).


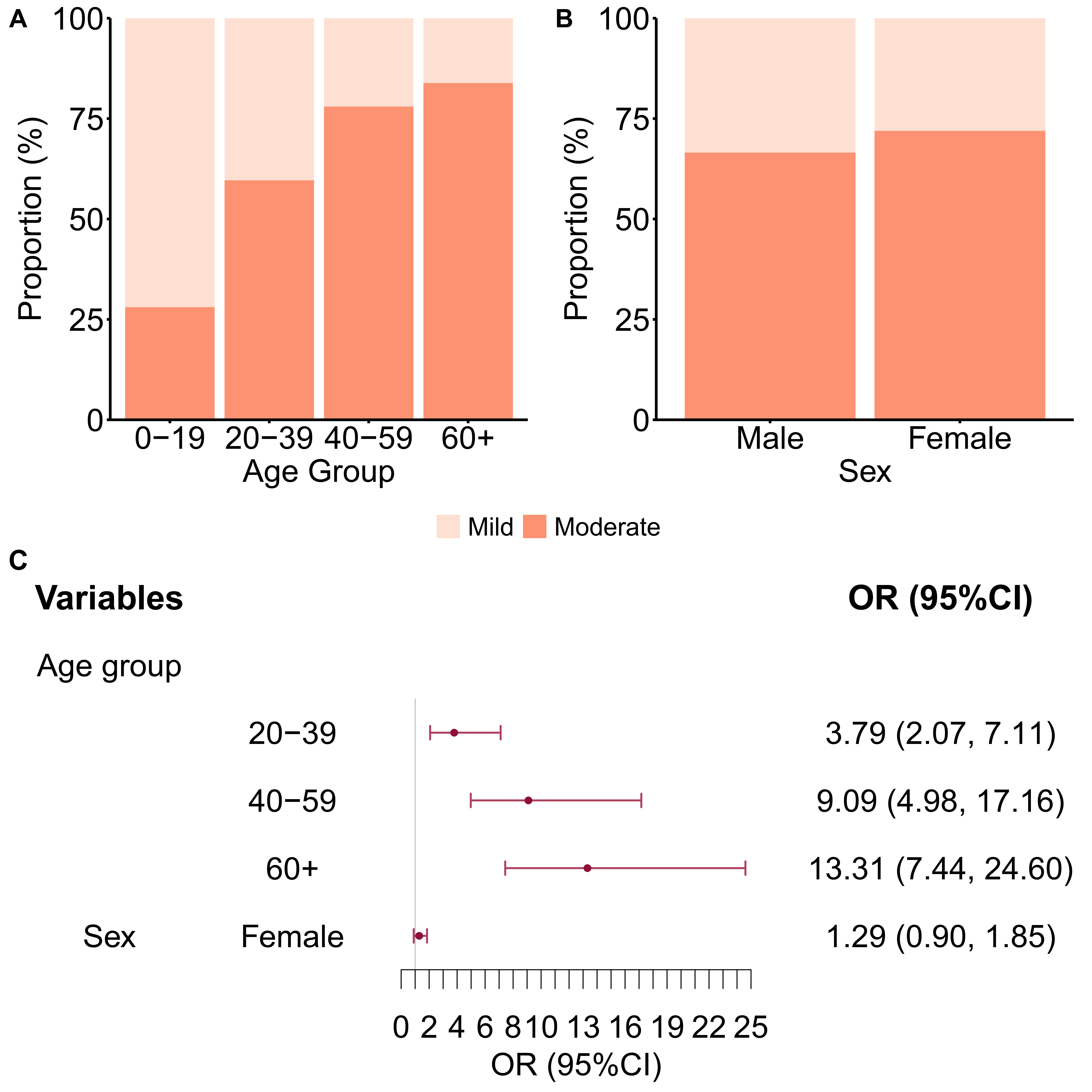
Figure S2. Univariate logistic regression analysis of the association of age groups (**A**) and sex (**B**) with clinical severity (mild or moderate) in patients from Yangzhou, and OR (95%CI) from the univariate logistic regression (**C**).


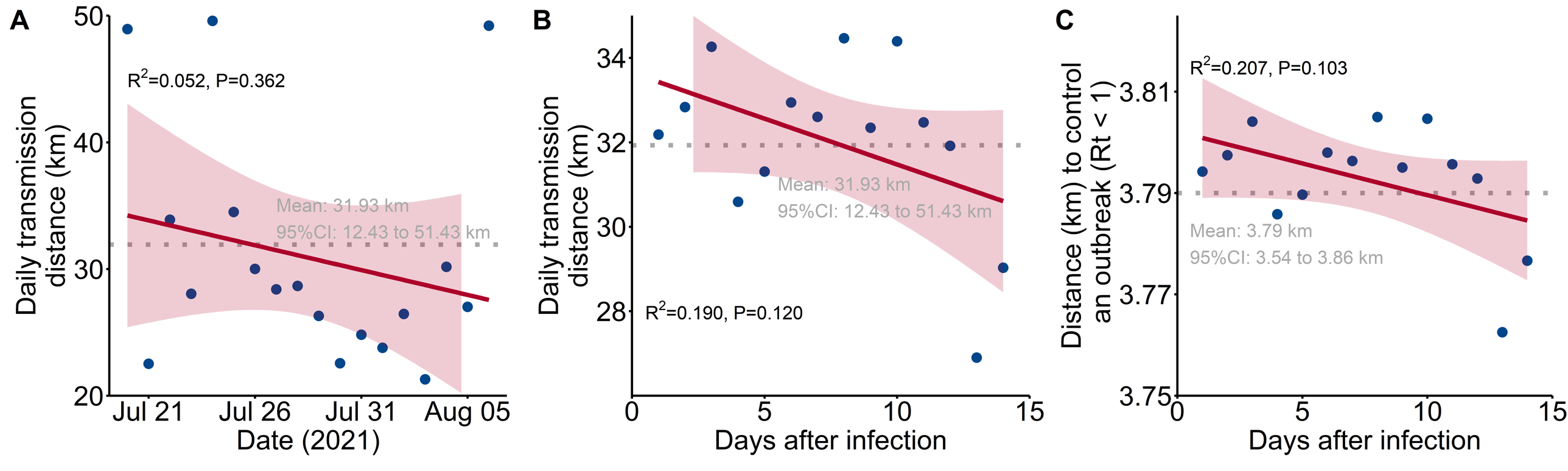
Figure S3. Daily transmission distance of the outbreak in Nanjing City, showing the association between daily transmission distance and date (**A**), daily transmission distance and days after infection (**B**), and distance used to control the outbreak and days after infection. The horizontal grey dotted lines are mean distances (31.93 km in **A** and **B**, 3.79 km in **C**).


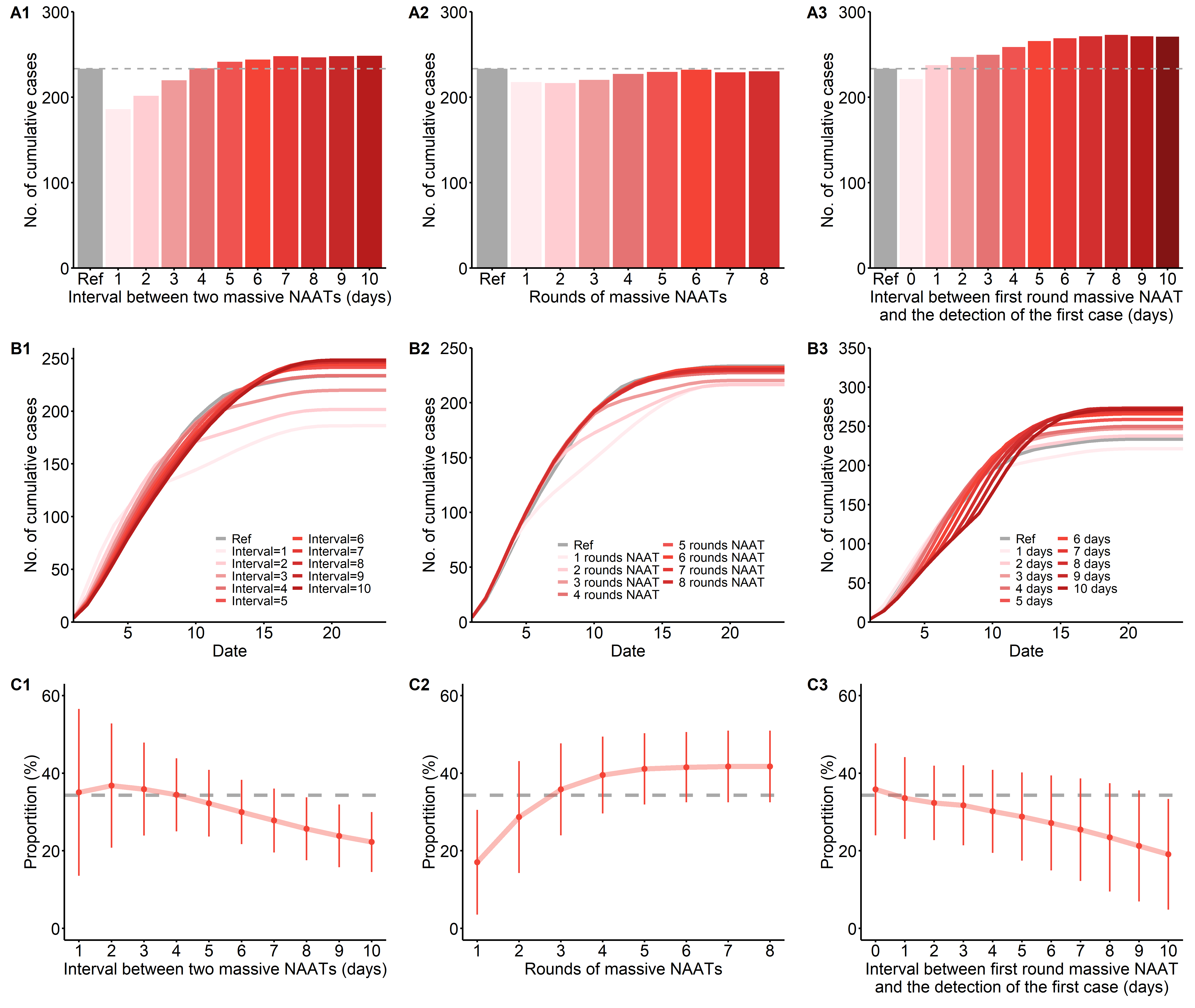
Figure S4. Number of cumulative cases (A and B) and percentage of cases found by massive NAAT (C) in Nanjing City for different intervals of NAAT (A1, B1, C1), different number of rounds of NAAT (A2, B2, C2), and different start time of NAAT (A3, B3, C3). The error bars in C, indicate 95% reference intervals.


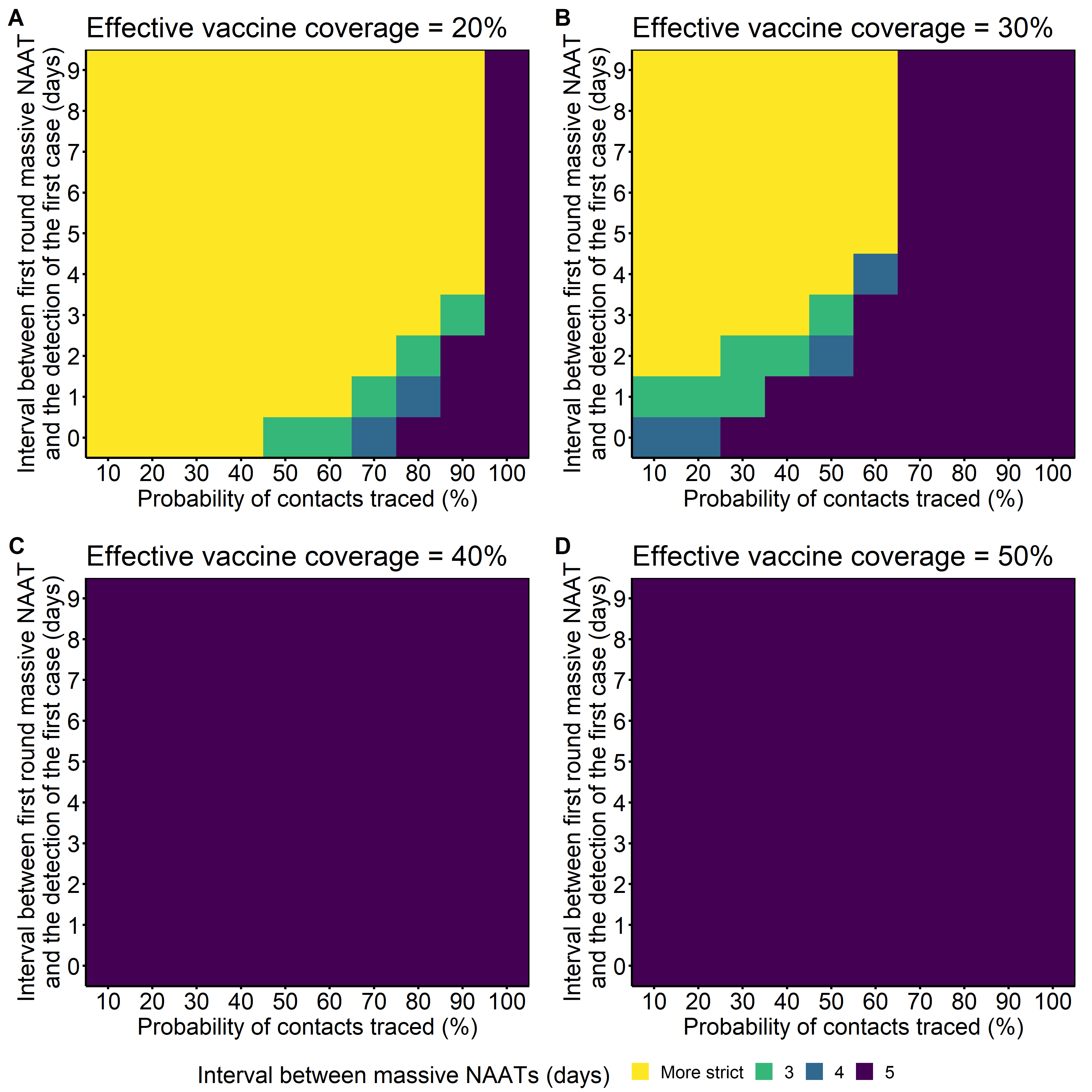
Figure S5. Combined effect of vaccine coverage, close contact tracing, and massive NAAT on limiting the cumulative number of cases in Nanjing City to 235. Each scenario had 3 rounds of NAAT.
